# Is Obturator Nerve Block Effective as Spinal Anesthesia in Preventing Adductor Spasms in General Anesthesia Without Muscle Relaxants?

**DOI:** 10.7759/cureus.22365

**Published:** 2022-02-18

**Authors:** Ihsan Guney, Guldeniz Argun

**Affiliations:** 1 Department of Anesthesiology and Reanimation, Adana City Training and Research Hospital, Adana, TUR; 2 Department of Anesthesiology and Reanimation, University of Health Sciences, Ankara Oncology Training and Research Hospital, Ankara, TUR

**Keywords:** obturator nerve block, spinal anesthesia, tur-b, laryngeal mask, general anesthesia practice

## Abstract

Aim: During transurethral resection of bladder tumor (TUR-BT), adductor muscle spasms in varying degrees can be seen due to stimulation of obturator nerve if the tumor is in the inferolateral localization. This can cause some serious complications such as bladder perforation. We aim to show the effectiveness of obturator nerve block (ONB) to avoid the adductor muscle spasm in general anesthesia applied with laryngeal mask (LMA) without using muscle relaxant according to the spinal anesthesia method.

Methods: The study has been designed prospectively and observationally. A total of 64 patients who underwent TUR-BT were divided into two groups. Group I consisted of 30 patients in whom TUR-BT was performed under general anesthesia without muscle relaxant + ONB. Group II consisted of 31 patients in whom TUR-BT was performed under spinal anesthesia + ONB. Intraoperative adductor spasm, the severity of adductor response, and surgeon satisfaction were recorded.

Results: Median values of adductor muscle strengths were found to be higher in Group I (p < 0.05). There was no statistically significant relationship between the anesthetic method and adductor spasm (p = 0.110). Of patients in Group I, 13.4% showed moderate or severe adductor response, whereas the ratio was 0% in Group II (p = 0.015). Surgeon satisfaction was similar in both groups (p = 0.363).

Conclusions: Obturator spasm was not different in both anesthesia techniques. General anesthesia without muscle relaxant combined with ONB was found effective to prevent adductor muscle spasms as the spinal anesthesia in TUR-BT operations. It has been concluded that surgical complications can be reduced via general anesthesia without the muscle relaxant method, although surgeons' satisfaction did not alter. General anesthesia and obturator block applications with the help of LMA without muscle relaxants can be preferred in short-term TUR-B operations where spinal anesthesia is not desired.

## Introduction

Transurethral resection of bladder tumor (TUR-BT) is a surgical method for both diagnosis and treatment of bladder tumor. Diagnostically, the main goal of TUR-BT is to obtain histological information about the morphological type of tumor and spread of disease. The main goal of treatment is the complete removal of all macroscopic noninvasive tumors [1].

The obturator nerve shows close proximity to the inferolateral bladder wall and the bladder neck in the pelvis. During transurethral resections of tumors in these localizations, electrical current through the resectoscope stimulates the obturator nerve. Although spinal anesthesia blocks the nerve's motor branch, the sensory branch may not be blocked. Violent adductor muscle spasms can be seen due to this stimulation. This situation is called obturator reflex and it generally causes involuntary movement of the legs (leg jerking). As a result, some serious undesirable consequences such as incomplete resection, bladder perforation, vascular injury, extravesical dissemination of cancer cells, uncontrollable bladder hemorrhage, and obturator muscle hematomas can happen [2]. Although obturator nerve stimulation is not rare, there is limited knowledge about the prevention of this condition in the literature. Many alternative methods such as resection under general anesthesia, partial retention of the bladder during resection, reduction of the intensity of the electrocautery, and alteration of electrical current polarity have been tried to prevent adductor muscle spasm so far [3,4]. However, all these techniques have not been found sufficiently effective in inhibiting obturator nerve stimulation [4].

Obturator nerve block (ONB) technique, first described by Prentiss et al. in 1965, is a regional anesthesia type that is easy to perform and has low complication rates [5]. The clinical necessity of obturator block has been better understood nowadays. The block can be ineffective if the nerve is placed deeply [3]. The induction of the obturator nerve due to failure of ONB during TUR-BT causes adductor muscle contraction and its complications [6,7]. A few different ONB techniques have been described and the effectiveness of these techniques has been searched so far [3,8]. Previous studies in the literature have shown that ONB can prevent adductor spasms and complications caused by it [1,6,9]. However, ONB alone is not sufficient in some cases, and more effective anesthetic techniques are still been investigated [1,10].

TUR-B operations are short-term operations. In general anesthesia applications, anesthesia with the help of a laryngeal mask (LMA) is frequently preferred without the use of muscle relaxants. In this study, in cases where spinal anesthesia is contraindicated or not requested by the patient or surgeon, we aimed to observe whether general anesthesia without muscle relaxant combined with ONB is effective to prevent adductor muscle spasm according to the spinal anesthesia in TUR-BT operations. To our knowledge, there were no other studies comparing these two methods in the literature.

## Materials and methods

After approval of the local ethics committee (protocol number: 25/7/2012-10.669) and written informed patient consent, a prospective observational study was designed from August 2012 to February 2013. Patients scheduled to undergo TUR-BT in our urology department because of deep-seated bladder tumor with lateral wall muscle invasion were evaluated. Patients with the anamnesis of advanced respiratory, cardiovascular disease, local anesthetic allergy, diabetes, peripheral neuropathy, neuromuscular disease, drug abuse, psychogenic diseases, previous surgery of the hip or inguinal region, coagulopathy, pre-existing obturator neuropathy, inguinal lymphadenopathy, perineal infection or hematoma at the needle insertion site, and having contraindications for regional anesthesia were excluded.

Sixty-four patients aged between 39 and 79 years were included in our study. We complied with the guidelines provided by the Consolidated Standards of Reporting Trials (CONSORT) and Strengthening the Reporting of Observational Studies in Epidemiology (STROBE). General anesthesia was applied with the aid of an LMA in cases where regional anesthesia was contraindicated or not preferred. These patients were classified as Group I (n = 30). Patients under spinal anesthesia were classified as Group II (n = 31). Sixty-four patients, 32 in each group, were included in the study. Two patients from Group I and one patient from Group II were excluded from the study because sufficient ONBs could not be obtained.

Preoperatively, prophylactic antibiotics (Cezol, cefazolin sodium, Deva Holding AS, Istanbul, Turkey) and premedication (0.05 mg/kg midazolam) were performed via a 20 G intravenous cannula. They were also pre-loaded with 500 ml of 0.9% normal saline intravenously. The operation times of both groups were recorded. Constant monitoring of electrocardiography (ECG), heart rate, and non-invasive blood pressure measurements were performed on all patients as standard anesthetic monitoring in the operation room. These data were recorded every 15 minutes during the operation.

ONB was performed on the right or left sides according to the tumor site. While the patients were in the supine position and their legs were in slightly (30°) abduction, their inguinal areas were cleaned with polyvinylpyrrolidone iodine before ONB. Local anesthesia of the intervention site was provided using 2 mL of 2% lidocaine. A peripheral nerve stimulator (Stimuplex®, B. Braun, Melsungen, Germany) and a 50 mm Teflon-insulated needle (22 G Stimuplex® A, B. Braun, Melsungen, Germany) were used. Obturator block was implemented with the help of high-frequency linear ultrasound (MyLab™Five, Esaote, Genova, Italy) and the interadductor method. The USG linear probe was placed 2 cm caudal and 2 cm medial to the femoral artery, felt over the inguinal ligament. The needle was inserted in-plane technique. The needle was advanced between the fascias of the adductor longus and adductor brevis muscles. Here the anterior branch of the obturator nerve is reached. Fasciculations were observed in the knee simultaneously by the Stimuplex®. Initially, a current of 2 mA at a frequency of 2 Hz was set. The current was reduced to 0.5 mA at a frequency of 2 Hz and 0.1 ms to achieve the intrinsic twitch response. Then 10 ml of 1% lidocaine was injected because it is known that the required lidocaine concentration for ONB should be over 1% to obtain an effective motor block (B20). The distribution of local anesthesia was observed between the two fasciae in ultrasound. The same procedure was performed between the fascias of the adductor brevis and adductor magnus muscles. Here, too, the posterior branch of the obturator nerve was blocked.

General anesthesia method

After applying ONB, induction of patients in Group I was performed with 1-2 μg/kg fentanyl and 2 mg/kg propofol. In anesthesia management of patients undergoing LMA, 1-2% sevoflurane and 33-67% O2/N2O were used. In this group, LMA was used without neuromuscular block.

Spinal anesthesia method

After applying ONB, spinal anesthesia was performed in the sitting position. Patients in Group II were given 0.5% hyperbaric bupivacaine 12.5 mg, with a 25 G spinal needle, entering from the L3-4 or L4-5 intervertebral spaces. Patients were placed in the supine position following drug administration. Upon reaching the level of Th10, which blocks conduction in the sensory nerve fibers of the bladder, the patients were placed in the lithotomy position.

Evaluation of anesthesia success of both groups

Before applying ONB, a sphygmomanometer that was inflated to 40 mmHg was placed between patients' legs while their knees were in extension. To evaluate the success of ONB in both groups, patients were requested to compress the sphygmomanometer. The power of the adductor muscle was recorded as mmHg. After performing ONB, the patients were requested to compress the sleeve of the sphygmomanometer between their legs with intervals of one minute. The maximum reduction in muscle strength was considered a success of ONB. The strength of the adductor muscle measured by sphygmomanometer was recorded preoperatively and after application ONB. The presence of intraoperative adductor spasm was also recorded and the degree of adductor response (mild, moderate, and severe) in patients with adductor spasm was evaluated. Since a possible adductor spasm would complicate the operation, the duration of the operation was also evaluated in both groups. Surgeon satisfaction (excellent, good, moderate, and poor) during the operation was recorded for the two different groups.

Statistical analysis

G*Power version 3.0.10 (Kiel University, Kiel, Germany) package program was used for determining sample size. Power analysis of the study showed that at least 30 patients in each group and at least 60 patients in total were needed to gain 90% power when the alpha error was set at 0.05, beta error at 0.10, and effect size at 0.95. A total of 64 patients, 32 in each group, were included in the study.

The normality of continuous variables was evaluated with the Shapiro-Wilk test. An independent sample t-test was used to compare parametric variables between the two groups, whereas the Mann-Whitney U test was used to compare nonparametric variables. In repeated measures, analysis of variance was used for comparing parametric variables. When differences were found, the post-hoc Bonferroni test was used to determine the source of the difference. Friedman's variance analysis was used for comparing non-parametric repeated variables. Bonferroni-corrected Wilcoxon test was used in post-hoc binary comparisons to assess the difference according to the results of the Friedman test. Comparisons between categorical variables were performed using chi-square analysis. Statistical analyses were performed by using IBM SPSS Statistics 21 (IBM Corp., Armonk, NY). A p-value of less than 0.05 was considered to be statistically significant.

## Results

A total of 61 patients (between 39 and 79 years) were included in the study. Of the patients, eight (13%) were females and 53 of them (87%) were males. While 30 (49%) patients underwent general anesthesia combined with ONB, 31 (51%) patients underwent spinal anesthesia combined with ONB. Demographic data are shown in Table 1.

**Table 1:** Distributions of gender and ASA score according to the groups. Group I: general anesthesia without muscle relaxant + obturator nerve block. Group II: spinal anesthesia + obturator nerve block. ASA, American Society of Anesthesiologists.

	Group I (n = 30)	Group II (n = 31)	P-value
Age (mean ± SD)	58 ± 10	62 ± 8	0.106
Gender			
Female (n, %)	3 (10)	5 (16.1)	0.476
Male (n, %)	27 (90)	26 (83.9)	
ASA score			
I (n, %)	6 (20)	3 (9.6)	0.365
II (n, %)	18 (60)	18 (58)	
III (n, %)	6 (20)	10 (32.4)	

In Group I, the median obturator block start time was 3.0 min, while it was 2.0 min in Group II. No statistical significance was found (p = 0.376). Before and after the ONB application, adductor muscle strengths were measured in two groups. Thus, it was evaluated whether effective ONB was obtained. Time-dependent changes of muscle strength were evaluated in both groups and this measurement was higher in the spinal anesthesia group (p < 0.001). The difference was significantly higher in Group II than Group I (p = 0.022 and p = 0.011, respectively).

**Figure 1:**
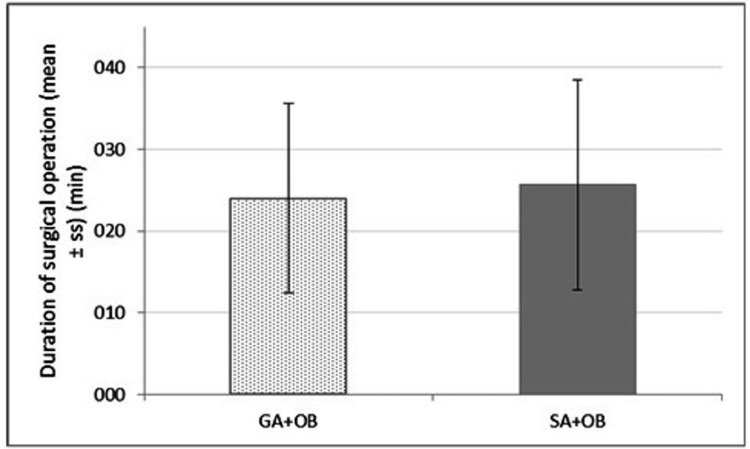
Mean surgical operation time (p = 0.37). Group I: general anesthesia without muscle relaxant + obturator nerve block. Group II: spinal anesthesia + obturator nerve block. GA, general anesthesia; SA, spinal anesthesia; OB, obturator nerve block.

**Figure 2:**
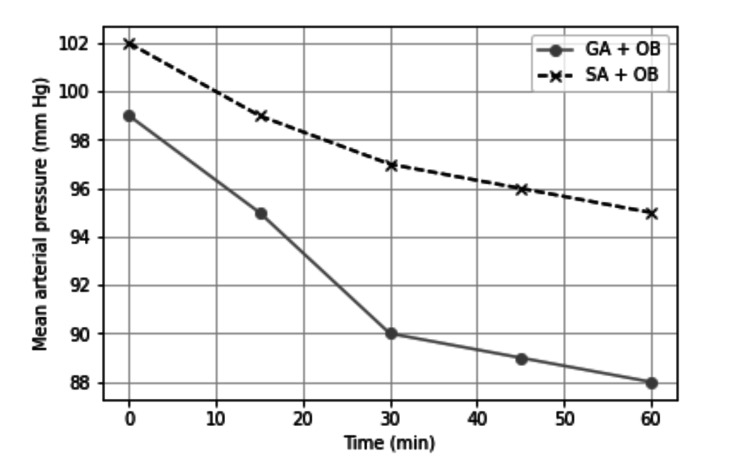
Mean arterial pressure (mmHg). Group I: general anesthesia without muscle relaxant + obturator nerve block. Group II: spinal anesthesia + obturator nerve block. GA, general anesthesia; SA, spinal anesthesia; OB, obturator nerve block.

**Figure 3:**
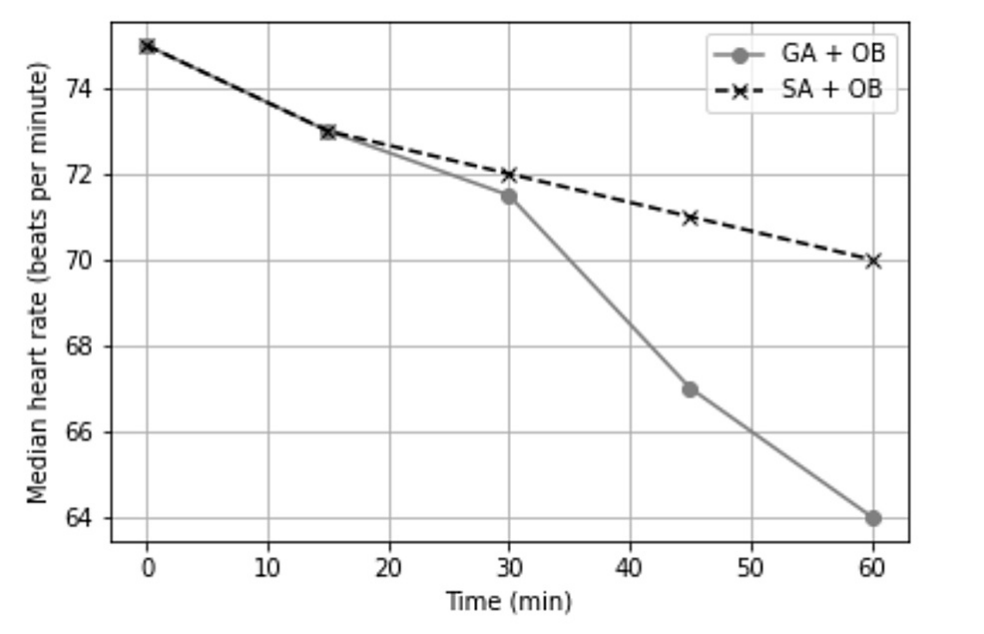
Median heart rate (p < 0.001). Group I: general anesthesia without muscle relaxant + obturator nerve block. Group II: spinal anesthesia + obturator nerve block. GA, general anesthesia; SA, spinal anesthesia; OB, obturator nerve block.

Distributions of adductor spasms, adductor responses, and surgeon satisfaction scores according to the two groups are demonstrated in Table 2. Adductor spasm rate was 13.7% in the general anesthesia group and 6.4% in the spinal anesthesia group, and the difference was statistically insignificant (p = 0.110). Moderate and severe adductor response was significantly higher in the general anesthesia group than in the spinal anesthesia group (p = 0.015). Mean surgical operation time and the data at 0, 15, 30, 45, and 60 minutes for mean arterial pressure and heart rates were not different between the groups (Figures 1-3).

**Table 2:** Distributions of adductor spasms, adductor responses, and surgeon satisfaction scores. Group I: general anesthesia without muscle relaxant + obturator nerve block. Group II: spinal anesthesia + obturator nerve block.

	Group I (n = 30)	Group II (n = 31)	P-value
Adductor spasm			
Yes (n, %)	4 (13)	2 (6.4)	0.110
No (n, %)	24 (80)	29 (93.6)	
Adductor response			
No (n, %)	24 (79.9)	29 (93.6)	0.015
Mild (n, %)	2 (6.7)	2 (6.4)	
Moderate (n, %)	2 (6.7)	-	
Severe (n, %)	2 (6.7)	-	
Surgeon satisfaction			
Bad (n, %)	-	-	0.363
Moderate (n, %)	-	-	
Good (n, %)	4 (13,3)	2 (6.4)	
Excellent (n, %)	26 (86.7)	29 (93.6)	

## Discussion

In this study, we aimed to compare whether the ONB could be effective in general anesthesia without muscle relaxants according to spinal anesthesia. We proved that in patients who cannot undergo spinal anesthesia due to technical difficulties and in cases in which using muscle relaxant is not desired, operations can be planned with the addition of ONB into general anesthesia without neuromuscular block.

Direct stimulation of the motor branch of the obturator nerve causes adduction. Some maneuvers such as partial retention of the bladder under general anesthesia, reduction of electrical current power, or alteration of electrical polarity during general anesthesia have been tried to prevent adductor muscle spasm during TUR-BT. But all these techniques have not been effective enough to prevent obturator nerve stimulation [11,12].

Prentiss et al. reported severe adductor muscle spasms in 20% of patients who underwent TUR-BT due to large tumors localized in the lateral bladder wall [5]. ONB was first proposed by Prentiss et al. in 1965 for the prevention of adductor spasms during TUR-BT [5]. They recommended ONB in addition to both general and spinal anesthesia. The other reported indications of ONB are the treatment of painful conditions in the hip joint and to relieve the pain secondary to adductor muscle spasm related to obturator neuralgia, multiple sclerosis, and paraplegia [9]. As a result, ONB has become increasingly popular in the following years. Atanassoff et al. compared ONB to three-in-one block to prevent adductor spasm during TUR-BT [6]. When spinal anesthesia was applied alone, the incidence of adductor spasms during TUR-BT was found to be higher (55%). Tatlısen et al. reported that the incidence of adductor spasm was 3% when they added ONB to spinal anesthesia [1]. In our study, we found that the incidence of adductor spasm was 6% in the spinal anesthesia + ONB group.

In a study by Patel et al., complete prevention of adductor spasm was reported as 96%, when they performed ONB combined with spinal anesthesia before TUR-BT. On the other hand, in the control group, in which ONB was not added to spinal anesthesia, surgeon satisfaction was not achieved in all cases during the operation. Bladder perforation occurred in two patients in the control group due to severe adductor spasm and urgent laparotomy was required in one of them [8]. So et al. observed adductor muscle spasm, which prevented their surgery, in a patient who underwent spinal anesthesia for TUR-BT. When they tried bilateral ONB combined with general anesthesia, the operation was successfully completed [13]. Darcın et al. found the obturator reflex rate higher in patients undergoing spinal anesthesia alone (83.3%) than those with ONB combined with spinal anesthesia (6.6%). In the combined group, the observed adductor response was mild [14].

The success rate of the classic approach of ONB is declared as about 50-91%. The main problem is difficulties in sensing the pubis tubercle in obese patients [15]. Because thick adipose tissue makes it difficult to see or palpate surface landmarks, the success rates of the inguinal approach of ONB in obese patients have reportedly decreased [14]. It is also an invasive approach, has technical difficulties, and requires a longer needle. As a result, it may increase the complication rates [3]. Therefore, the inguinal approach of ONB was described as an alternative, superficial, and comparatively easier method and it was highlighted as having lower complication rates [16]. But the main limitation of this technique is the lack of access to the obturator nerve branches originating from the hip joint fossa. Nonetheless, some studies found higher success rates via the inguinal approach rather than the classic approach (97.1% vs. 71.4%) [3,4,8]. Moreover, surgeon satisfaction was higher in the inguinal approach of ONB due to lack of leg jerking and less complication rates [4]. Onset time of the block was also reported more rapid in the inguinal approach by Aghamohammadi et al. [4]. Conversely, there were no significant differences between the success rates of the two modalities according to Moningi et al. [3]. In our study, we encountered only one failed block case.

We used an LMA without neuromuscular block in patients who underwent general anesthesia. Although previous studies have reported that succinylcholine and tubocurarine were used to inhibit adductor spasms during general anesthesia [3,13,17], some studies have revealed opposed results, and these anesthetic agents were found ineffective [1,8,9,13]. Spinal anesthesia has been frequently preferred to general anesthesia in TUR-BT operations because the majority of patients undergoing TUR-BT are older and they have more concomitant systemic diseases [18]. For that reason, spinal anesthesia is more advantageous than general anesthesia in preventing intraoperative and postoperative complications. However, it is known that spinal anesthesia alone is not enough to prevent adductor spasms and obturator reflex [19]. Inadequate response to the obturator block added in spinal anesthesia or general anesthesia with muscle relaxants can be attributed to the invasiveness of the tumor or the presence of an accessory obturator nerve branch.

In our study, Labat's classical technique was used for ONB using a nerve stimulator, and 95% of the patients achieved adequate nerve block. This technique is the most commonly used method but the previous studies have also reported that similar success rates of ONB were obtained using different techniques such as interadductor, inguinal, or intravesical approaches [3,20,21]. Wassef et al. identified the interadductor approach as a more practical method [20]. Although they stated that patient satisfaction rates were low in the classical method, we did not encounter any negative conditions related to patient dissatisfaction during and after the classic method. The reported success rate was 60% in the intravesical approach, so it has not been widespread [21]. In the literature, the presence of accessory obturator nerve has been reported as a factor that limits the success of ONB. The accessory obturator nerve is formed by the fusion of anterior branches of lumbar 3 and 4 [22]. The accessory obturator nerve follows a different pathway from the obturator nerve and limits the effectiveness of ONB unless it is blocked [22,23]. On the other hand, the accessory obturator nerve is accompanied by the obturator nerve at the ratio of 10-30% (18). In our study, adductor spasm was seen less (6.4%) in the combined method with spinal anesthesia. We think that the failure rate due to unblocked accessory obturator nerve is not common.

In our study, among patients who showed adductor muscle spasms, more severe adductor muscle responses were seen in the group undergoing combined with general anesthesia. The combined method with spinal anesthesia was found more successful in terms of preventing obturator spasms. The success rate was higher in the latter method (93.6% vs. 80%). Our success rates for the combined method with spinal anesthesia were in line with the previous reports.

Although predicting the incidence of adductor muscle spasm during TUR-BT is difficult because of anesthesia technique, surgical technique, and tumor localization, especially in cases of tumors localized in the inferolateral bladder wall, the higher rates of obturator reflex have been reported until now [3]. In this study, we aimed to investigate the efficacy of ONB that was applied before general or spinal anesthesia in patients undergoing TUR-BT. Although there were no significant differences in terms of adductor muscle spasm and surgeon satisfaction, when adductor spasm occurred, the patients' adductor responses were more severe in the combined general anesthesia group.

Our main limitation is the low patient population and lack of a control group. We did not calculate the incidence rates of obturator reflex during TUR-BT because we did not have a control group (patients not undergoing ONB). We used general anesthesia without neuromuscular block in Group I. Lower success rates in the prevention of obturator reflex can be attributed to the lack of neuromuscular block. Another group in which ONB is combined with general anesthesia added to the neuromuscular block can be formed. Because our results belonged to a single center, there is still a need for multicenter studies with larger patient populations.

## Conclusions

In this study, it was determined that ONB combined with spinal anesthesia in TUR-BT operations was more efficient in terms of the severity of the adductor responses. Although more severe adductor muscle responses can be seen in cases in which spinal anesthesia is contraindicated, in patients who cannot undergo spinal anesthesia due to technical difficulties and in cases in which using muscle relaxant is not desired, operations can be planned with the addition of ONB into general anesthesia without neuromuscular block. Obturator spasm was not different in both anesthesia techniques. General anesthesia without muscle relaxant combined with ONB was found effective in preventing adductor muscle spasms as the spinal anesthesia in TUR-BT operations. It has been concluded that surgical complications can be reduced via general anesthesia without the muscle relaxant method, although surgeons’ satisfaction did not alter. General anesthesia and obturator block applications with the help of LMA without muscle relaxants can be preferred in short-term TUR-B operations where spinal anesthesia is not desired.
